# Association Between Admission Eosinophil Count and Hospital Resource Use in Community-Acquired Pneumonia: A Prospective Multicenter Study

**DOI:** 10.1016/j.chest.2026.03.018

**Published:** 2026-03-31

**Authors:** Barbara Christine Weckler, Qiong Wu, Wilhelm Bertrams, Max Kutzinski, Rieke Reiter, Saeed A. Khan, Peter Ahnert, Norbert Suttorp, Martin Witzenrath, Christian Wildberg, Mathias W. Pletz, Jan Rupp, Ulrich M. Zissler, Timm Greulich, Thomas Brenner, Peter Alter, Gernot Rohde, Claus Franz Vogelmeier, Andreas Mayr, Bernd Schmeck

**Affiliations:** a Department of Respiratory, Intensive Care, and Sleep Medicine, Clinic for Respiratory Infections, Marburg University, Marburg, Germany; b Institute for Lung Research, Universities of Giessen and Marburg Lung Center (UGMLC), Marburg University, Marburg, Germany; c Institute for Medical Biometry and Statistics, Marburg University, Marburg, Germany; d Institute of Laboratory Medicine, Marburg University, Marburg, Germany; e Department of Geography, Marburg University, Marburg, Germany; f Institute for Medical Informatics, Statistics and Epidemiology, University of Leipzig, Leipzig, Germany; g German Center for Lung Research (DZL), Leipzig, Germany; h Department of Infectious Diseases, Respiratory Medicine and Critical Care, Charité-Universitätsmedizin Berlin, Corporate Member of Freie Universität Berlin and Humboldt-Universität zu Berlin, Berlin, Germany; i German Center for Lung Research (DZL), Berlin, Germany; j CAPNETZ STIFTUNG, Hannover, Germany; k Institute for Infectious Diseases and Infection Control and Center for Sepsis Care and Control, Jena University Hospital, Friedrich-Schiller-University, Jena, Germany; l Infectious Diseases Clinic and Institute of Medical Microbiology, University Hospital Schleswig-Holstein, Campus Luebeck, Luebeck, Germany; m Technology Transfer Center for Building Biology, Airway and Indoor Health, Rosenheim Technical University of Applied Sciences, Freilassing, Germany; n Department of Medicine, Pulmonary and Critical Care Medicine, University Medical Center Giessen and Marburg, Marburg University, German Center for Lung Research (DZL), Universities of Giessen and Marburg Lung Center (UGMLC), Marburg, Germany; o Department of Pediatrics, Klinikum rechts der Isar, School of Medicine, Technical University of Munich (TUM), Germany; p Department of Respiratory, Intensive Care, and Sleep Medicine, Marburg University, Marburg, Germany; q German Center for Lung Research (DZL), Marburg, Germany; r Biomedical Research in Endstage and Obstructive Lung Disease Hannover (BREATH), Member of the German Center for Lung Research (DZL); s Core Facility Flow Cytometry – Bacterial Vesicles, Marburg University, Marburg, Germany; t Institute for Lung Health (ILH), Giessen, Germany; u German Center of Infectious Disease Research, Marburg, Germany; v Center for Synthetic Microbiology (Synmikro), Marburg University, Marburg, Germany

**Keywords:** biomarker, eosinophil count, health care resources, pneumonia

## Abstract

**Background:**

Eosinopenia has been associated with adverse outcomes in community-acquired pneumonia (CAP). However, its relationship with hospital resource use remains unclear.

**Research Question:**

What is the association between admission eosinophil counts and hospital resource use among adults with CAP?

**Study Design and Methods:**

This prospective multicenter cohort study (Community-Acquired Pneumonia Network of Competence [CAPNETZ]; Identifier: 2024-07-11-CHV6) has enrolled patients ≥ 18 years of age with CAP in university hospitals in Germany since 2017. Associations between admission blood eosinophil counts and hospital resource use—ICU admission, mechanical ventilation, and length of stay—were assessed using multivariable regression models. The optimal eosinophil count threshold for stratifying patients by ICU admission and mechanical ventilation rates was identified, and outcomes were compared between patients above and below this threshold.

**Results:**

Lower eosinophil counts at admission were associated with increased ICU admission (n = 1,639; *P* < .001), including among patients treated with systemic glucocorticoids (*P* = .002) and those not receiving glucocorticoids (*P* = .047). Lower eosinophil counts also were associated with higher rates of mechanical ventilation (*P* = .014) and longer hospital stays (*P* = .024). An eosinophil count threshold of 10 cells/μL was identified as the cutoff that best distinguished patients with higher vs lower risk of ICU admission and mechanical ventilation. Patients with eosinopenia (≤ 10 cells/μL) showed higher ICU admission rates (14.2% vs 8.5%; *P* < .001; adjusted OR, 1.78), increased mechanical ventilation rates (9.1% vs 5.2%; *P* = .003; adjusted OR, 1.82), and longer hospitalization (mean, 10.2 days vs 9.0 days; *P* = .013).

**Interpretation:**

Our results show that admission eosinopenia (≤ 10 cells/μL) was associated with greater hospital resource use and may serve as a practical biomarker for health care resource planning.

**Clinical Trial Registration:**

German Clinical Trials Register; No.: DRKS00005274; URL: https://drks.de/

FOR EDITORIAL COMMENT, SEE PAGE 285

**Take-Home Points**

**Research Question:** Is admission blood eosinophil count associated with increased hospital resource use in adults hospitalized with community-acquired pneumonia (CAP)?

**Results:** Lower eosinophil counts, particularly values of ≤ 10 cells/μL, were associated with greater hospital resource use, including higher ICU admission rates, increased mechanical ventilation needs, and longer hospital stays.

**Interpretation:** Our results show admission eosinopenia may serve as an early and easily obtainable biomarker to guide hospital resource planning in patients with CAP.

Lower respiratory tract infections, including community-acquired pneumonia (CAP), remain among the top causes of morbidity and mortality,^1^ accounting for 2.5 million deaths annually. Incidence rates of CAP vary widely. In Europe, the incidence ranges from 20.6 to 79.9 cases per 10,000 person-years.^2^^,^^3^ In the United States, the incidence spans 24.8 to 106 cases per 10,000 person-years for adults younger than 65 years, increasing to 164.3 cases per 10,000 person-years for those older than 80 years.^4^^,^^5^ Hospitalization costs also escalate with age, with mean costs of approximately €8,500 for patients 65 to 74 years of age and €17,000 for those 85 years of age and older.^6^ Collectively, CAP imposes a major clinical and economic strain on health care systems globally.^1^, ^2^, ^3^, ^4^, ^5^, ^6^, ^7^, ^8^, ^9^

Despite this burden and the existence of established severity scores, simple, routinely available biomarkers for predicting hospital resource requirements on admission, such as ICU admission, mechanical ventilation, and length of stay, could supplement existing risk stratification tools. Emerging evidence suggests that peripheral eosinophil counts may predict adverse outcomes independently in CAP.^10^^,^^11^ However, the usefulness of eosinopenia as an early, practical biomarker to inform resource allocation has not yet been analyzed systematically. Early and accurate identification of patients who are likely to require intensive care or prolonged hospital stays may enable more efficient hospital resource use and improved clinical outcomes, a pressing priority given the global strain on health care systems.^12^ In this study, we investigated the association between admission eosinophil counts and hospital resource use in adults with CAP.

## Study Design and Methods

We conducted a prospective, multicenter observational study within the Community-Acquired Pneumonia Network of Competence (CAPNETZ) network (Identifier: 2024-07-11-CHV6; German Clinical Trials Register Identifier: DRKS00005274) at university hospitals across Germany.^13^ The CAPNETZ study protocol was approved by the ethics committee of the Hannover Medical School (Identifier: 301-2008).

Eligible patients were ≥ 18 years of age, had radiographically confirmed pulmonary infiltrates, and demonstrated at least 1 of the following: fever (≥ 38.3 °C), cough, purulent sputum, or focal chest findings on auscultation. Exclusion criteria were acquired or drug-induced immunodeficiency, active TB, or suspected nosocomial pneumonia.

At admission, clinical history and vital signs were recorded using a standardized protocol. Patients were followed up throughout their hospital stay, and all clinical data and outcomes were recorded in a centralized electronic database. Written informed consent was obtained from all participants before enrolment. The study was approved by local ethics committees at each participating center.

Data collected at hospital admission included age, sex, laboratory parameters (eosinophil count, lymphocyte count, neutrophil count, creatinine, C-reactive protein [CRP], and hematocrit), pneumonia severity scores (confusion, urea, respiratory rate, BP, and age ≥ 65 years [CURB-65]; confusion, urea, respiratory rate, and BP [CURB]; and confusion, respiratory rate, BP, and age ≥ 65 years [CRB-65]), preexisting comorbidities, and use of systemic (oral or IV) or nonsystemic (inhaled, intranasal, or rectal) glucocorticoid therapy. Outcomes included ICU admission, need for noninvasive or invasive mechanical ventilation, ICU length of stay, duration of mechanical ventilation, total hospital length of stay, and in-hospital mortality.

The primary end point was ICU admission. Secondary end points included: (1) need for mechanical ventilation (defined herein), (2) ICU length of stay, (3) duration of mechanical ventilation, (4) total hospital length of stay, and (5) in-hospital mortality. Invasive mechanical ventilation was defined as endotracheal intubation or tracheostomy with positive pressure ventilation, and noninvasive mechanical ventilation was defined as CPAP or bilevel positive airway pressure for acute respiratory failure related to pneumonia. Preexisting long-term nocturnal noninvasive positive pressure ventilation was not classified as mechanical ventilation. All end points were assessed from admission until discharge or in-hospital death. Patients missing eosinophil counts or key covariates (age, sex, creatinine, CRP, and hematocrit) were excluded from the respective multivariable analyses. Descriptive statistics were summarized as median or mean values with interquartile ranges (interquartile ranges [IQRs]; 25th and 75th percentiles), and categorical variables were summarized as frequencies and percentages.

We analyzed associations between admission eosinophil counts (modeled as a continuous variable) and hospital resource use outcomes using multivariable generalized regression models. Covariates included age, sex, creatinine, CRP, and hematocrit. Analyses were performed for the overall cohort and stratified by glucocorticoid exposure (systemic therapy vs no therapy). Additionally, the associations between eosinophil counts and pneumonia severity scores (CURB-65, CURB, and CRB-65) were examined.

Receiver operating characteristic curve analysis was performed to identify the optimal eosinophil count threshold for predicting ICU admission and mechanical ventilation using Youden’s index. Sensitivity, specificity, and predictive values were calculated with 95% CIs. To assess robustness, 100 bootstrap samples were generated; for each, the optimal eosinophil threshold and area under the receiver operating characteristic curve were calculated and compared with estimates from the original data set.

Patients then were stratified into eosinopenia (eosinophil count less than or equal to optimal threshold) and no eosinopenia (eosinophil count more than optimal threshold) groups. Baseline laboratory parameters and comorbidities were compared using the Mann-Whitney *U* test and Fisher exact test, respectively. Outcomes, including ICU admission, mechanical ventilation, ICU and hospital lengths of stay, and ventilation duration, were compared between groups using multivariable logistic or linear regression models adjusted for age, sex, creatinine, CRP, and hematocrit. In-hospital mortality was compared between groups.

An additional analysis examined associations between eosinopenia, lymphocytopenia, and their combination with hospital resource use. Lymphopenia was defined as an absolute lymphocyte count of < 0.724 cells/nL, as established by Bermejo-Martín et al.^14^ Three binary classification markers were created: eosinopenia (≤ 10 cells/μL, based on the receiver operating characteristic curve-derived cutoff), lymphopenia (< 0.724 cells/nL), and dual cytopenia (defined as the presence of both eosinopenia and lymphopenia at hospital admission). Multivariable logistic regression models adjusted for age, sex, creatinine, CRP, and hematocrit were used to examine the association between each cytopenia category—and their combination—and ICU admission, mechanical ventilation, and hospital length of stay. Predictive performance was evaluated using Youden’s index.

As a sensitivity analysis, inverse probability weighting (IPW) was performed to adjust for confounding between the eosinopenia (≤ 10 cells/μL) and no eosinopenia (> 10 cells/μL) groups. Propensity scores, which represent the probability of belonging to the eosinopenia group, were estimated via logistic regression using admission variables with minimal missingness, including age, sex, creatinine, CRP, hematocrit, comorbidities, preadmission glucocorticoid exposure, and pneumonia severity scores (CURB-65 and CRB-65). IPW analyses were restricted to patients with complete data for all variables. Weights were computed as 1 / propensity score for the eosinopenia group and 1 / (1 − propensity score) for the no eosinopenia group. Balance diagnostics were performed using standardized mean differences, with standardized mean differences of < 10% considered adequate for covariate balance. Weighted generalized regression models then were applied to study the associations between continuous eosinophil counts and both ICU admission and mechanical ventilation.

As another sensitivity analysis, multiple imputation by chained equations was performed to address missing data, assuming that data were missing at random. The imputation model included all variables from the main analyses: ICU admission, mechanical ventilation, eosinophil count, age, sex, CRP, creatinine, and hematocrit. Imputed data sets were analyzed using the same multivariable regression models as in the primary analysis. Adjusted ORs and adjusted mean differences were reported with 95% CIs. *P* values of < .05 were considered statistically significant. All analyses were conducted in R version 4.4.2 software (R Foundation for Statistical Computing).

## Results

### Patient Characteristics of the Entire Cohort

The study enrolled 2,880 patients (63% male patients; median age, 68 years). Among these, 11.1% of patients (n = 321) were admitted to the ICU, 6.9% of patients (n = 200) received mechanical ventilation, and 9.1% of patients (n = 261) died during hospitalization. Baseline characteristics of the full cohort are shown in Table 1. Of all participants, 1,639 participants had complete data for all covariates and were included in the multivariable analyses.

**Table 1:** Characteristics of the Entire Cohort

Variable	Overall Group (N = 2,880)
Age, y (N = 2,880 [100.0%])	68 (56-78)
Male sex (N = 2,880 [100.0%])	1,811 (62.9)
Weight, kg (n = 2,688 [93.3%])	76.45 (65-90)
BMI, kg/m^2^ (n = 2,664 [92.5%])	25.7 (22.8-29.5)
Eosinophil count, /μL (n = 1,673 [58.1%])	38.56 (0-136)
Creatinine, mg/dL (n = 2,827 [98.2%])	0.98 (0.78-1.25)
Hematocrit, % (n = 2,833 [98.4%])	37 (33-40.7)
CRP, mg/dL (n = 2,816 [97.8%])	11.42 (4.96-19.96)
ICU admission rate (n = 2,880 [100.0%])	321 (11.1)
Mechanical ventilation rate (n = 2,880 [100.0%])	200 (6.9)
In-hospital mortality (n = 2,880 [100.0%])	261 (9.1)
ICU length of stay, d (n = 321 [11.1%])	3 (2-7)
Length of mechanical ventilation, d (n = 200 [6.9%])	3 (1-9)
Length of hospital stay, d (n = 2,880 [100.0%])	7 (5-11)

Data are presented as No. (%) or median (interquartile range). CRP = C-reactive protein.

### Association Between Eosinophil Counts and ICU Admission, Mechanical Ventilation, Hospital Length of Stay, CURB-65, CURB, and CRB-65

**Figure 1:**
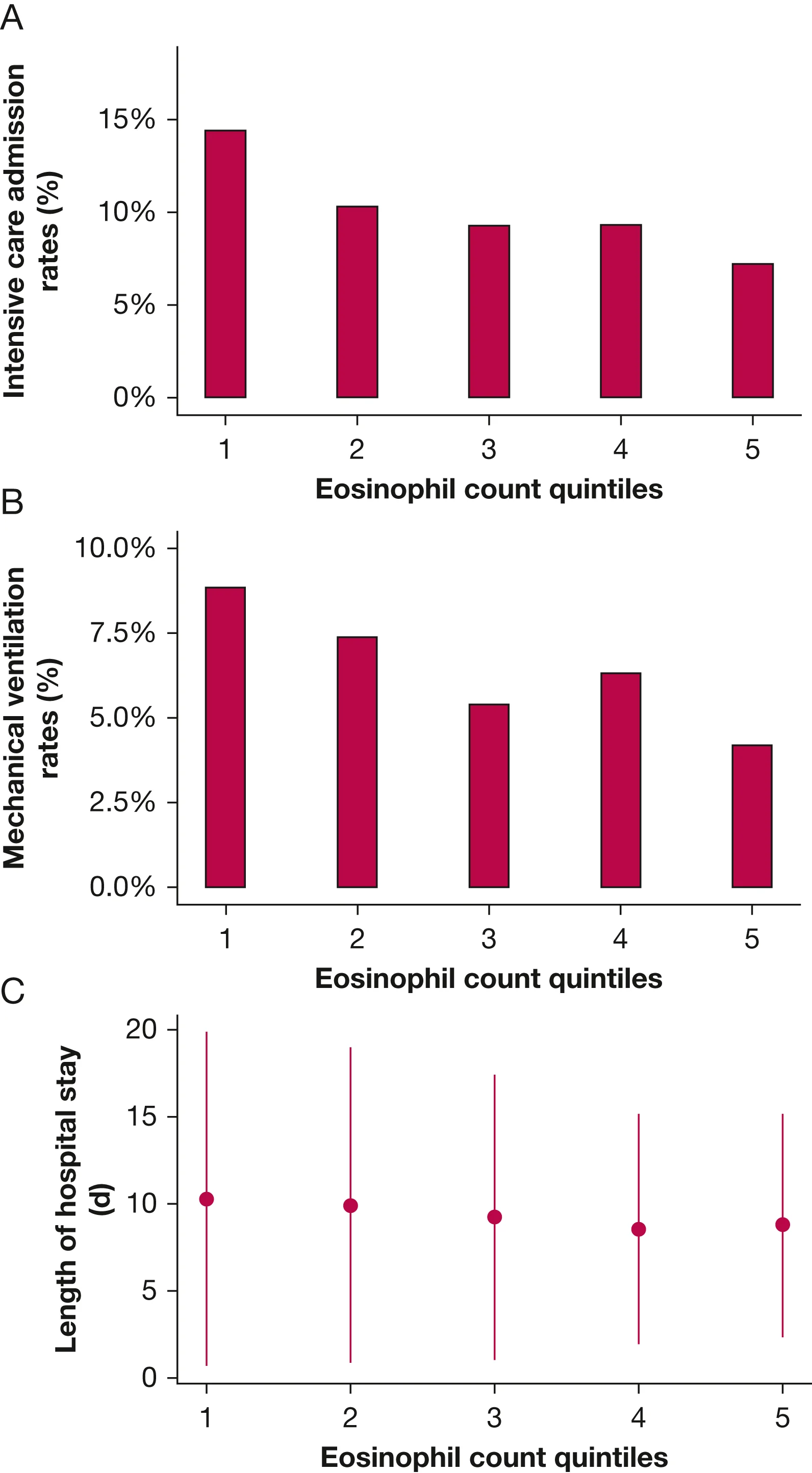
A, Bar graph showing crude ICU admission proportions (%) across eosinophil count quintiles at hospital admission. Statistical inference is based on these adjusted model estimates. This figure is provided to illustrate the unadjusted pattern. B, Bar graph showing crude mechanical ventilation proportions (%) across eosinophil count quintiles at hospital admission. Statistical inference is based on these adjusted model estimates. This figure is provided to illustrate the unadjusted pattern. C, Bar graph showing crude median length of hospital stay (in days) across eosinophil count quintiles at hospital admission. Statistical inference is based on these adjusted model estimates. This figure is provided to illustrate the unadjusted pattern.

In multivariable regression analyses (n = 1,639) (Fig 1A), lower eosinophil counts at hospital admission were associated with higher odds of ICU admission after adjustment for age, sex, creatinine, C-reactive protein, and hematocrit (adjusted OR per 1-unit decrease in log-transformed eosinophil count, 1.04; 95% CI, 1.02-1.06; *P* < .001). This association remained significant among patients who had received systemic glucocorticoids (n = 375; adjusted OR per 1-unit decrease in log-transformed eosinophil count, 1.07; 95% CI, 1.02-1.11; *P* = .002) and among those without glucocorticoid treatment (n = 1,228; adjusted OR per 1-unit decrease in log-transformed eosinophil count, 1.03; 95% CI, 1.00-1.06; *P* = .047). Among patients treated with systemic glucocorticoids, 62 of 375 patients (16.5%) required ICU admission, compared with 106 of 1,228 patients (8.6%) among those not treated with glucocorticoids. Lower eosinophil counts also were associated significantly with increased mechanical ventilation rates (n = 1,639; adjusted OR per 1-unit decrease in log-transformed eosinophil count, 1.04; 95% CI, 1.01-1.07; *P =* .014) (Fig 1B) and longer hospital stays (n = 1,636; increase in log-transformed days in hospital per 1-unit decrease in log-transformed eosinophil count, 0.006; 95% CI, 0.0008-0.012; *P =* .024) (Fig 1C). Back-transformation of the hospital stay coefficient yields a 1.4% increase in hospital length of stay (approximately 0.13 days for a 9-day baseline) for a 10-fold decrease in admission eosinophil count. No significant associations were observed between eosinophil counts and ICU length of stay (n = 321; increase in log-transformed ICU days per 1-unit decrease in log-transformed eosinophil count, 0.015; 95% CI, –0.013 to 0.043; *P =* .298) or duration of mechanical ventilation (n = 200; increase in log-transformed days of mechanical ventilation per 1-unit decrease in log-transformed eosinophil count, 0.031; 95% CI, –0.010 to 0.073; *P* = .139). Lower eosinophil counts were associated significantly with higher CURB-65 (n = 1,295; coefficient, –0.01; *P* = .016) and CURB (n = 1,295; coefficient, –0.01; *P* < .001) values, respectively. The association between eosinophil count and CRB-65 value was not significant (n = 1,495; coefficient, 0.00; *P* = .331).

### Threshold Analysis for Eosinopenia

**Figure S1:**
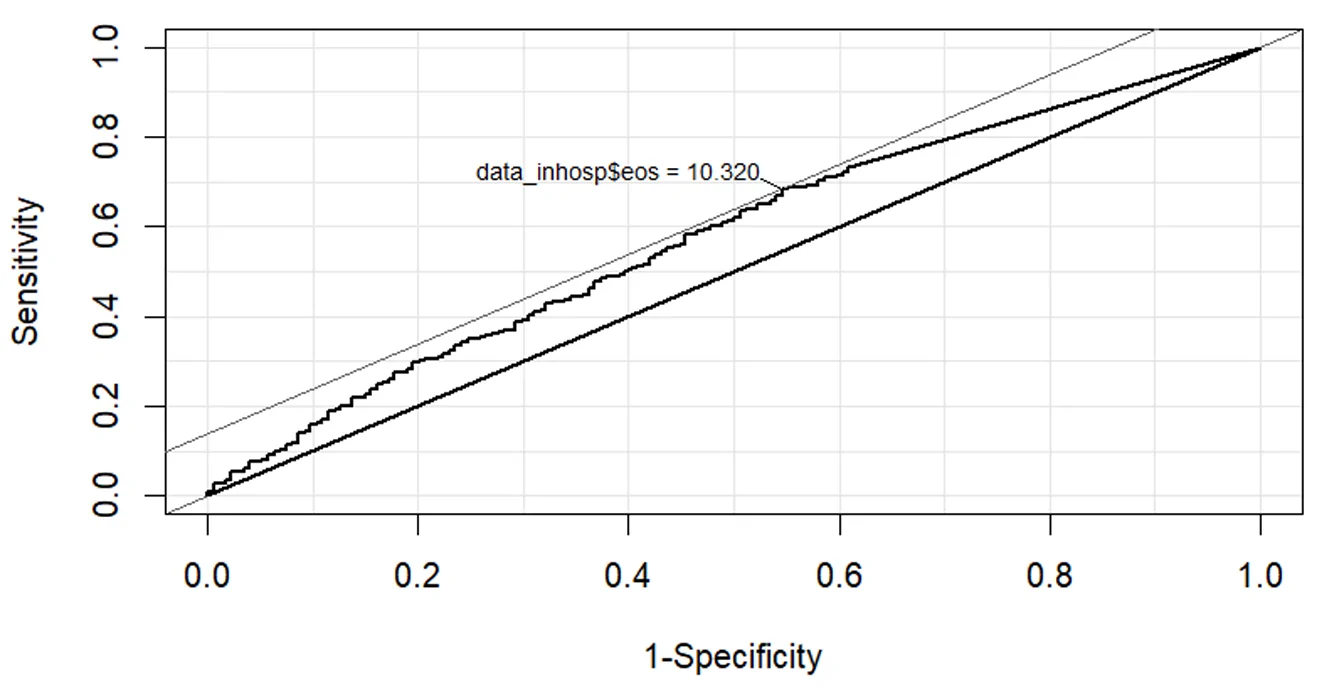


**Figure S2:**
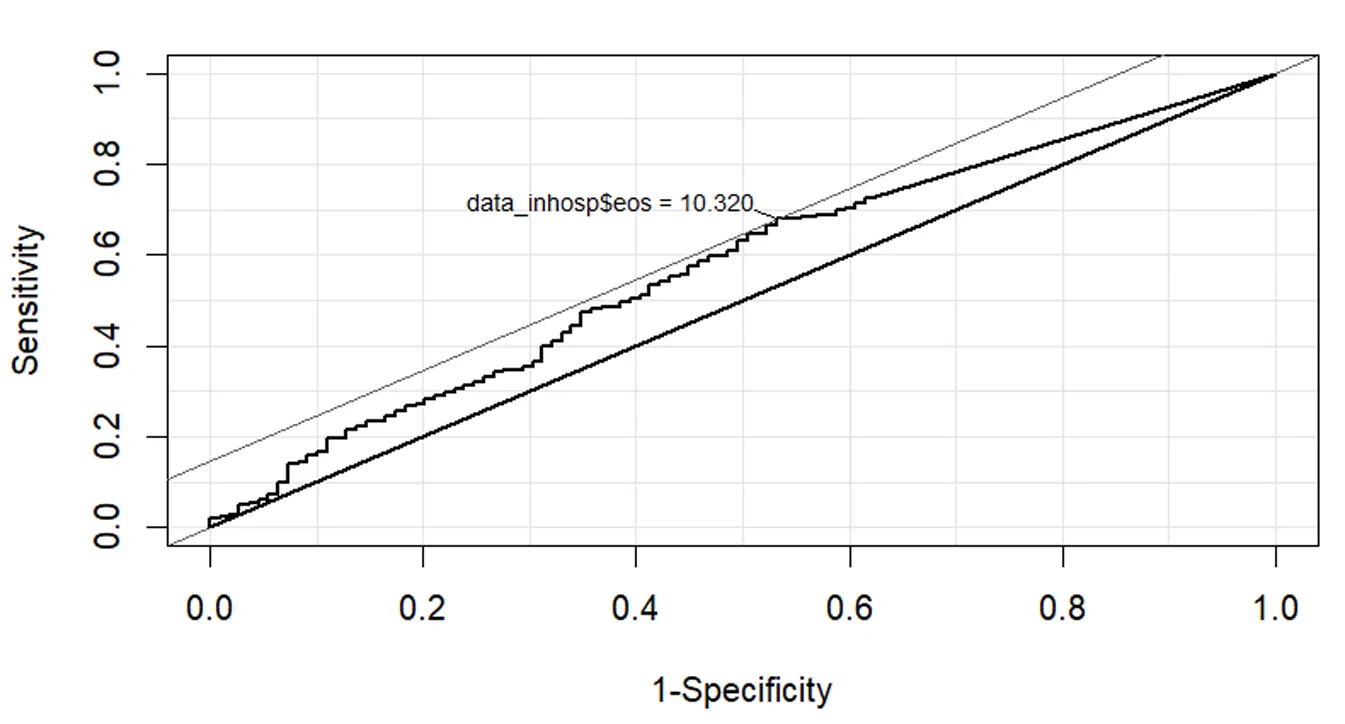


An eosinophil count threshold of ≤ 10 cells/μL optimally stratified risk of ICU admission and mechanical ventilation. At this threshold, negative predictive values were 91.5% for ICU admission and 94.8% for mechanical ventilation. Sensitivity, specificity, and positive predictive value were 45.4%, 68.4%, and 14.3%, respectively, for ICU admission risk (Table 2, e-Fig 1), and 46.8%, 67.9%, and 9.2%, respectively, for mechanical ventilation risk (Table 2, e-Fig 2).

**Table 2:** Sensitivity, Specificity, and Positive and Negative Predictive Values for an Eosinophil Count Threshold of ≤ 10/μL

Metric	ICU Admission Risk, % (95% CI)	Mechanical Ventilation Risk, % (95% CI)
Negative predictive value	91.5 (89.7-93.1)	94.8 (93.4-96.0)
Sensitivity	45.4 (37.9-53.1)	46.8 (37.2-56.6)
Specificity	68.4 (66.0-70.7)	67.9 (65.5-70.2)
Positive predictive value	14.3 (11.5-17.5)	9.2 (6.9-11.9)

Bootstrap validation confirmed that the eosinophil count threshold used in the primary analysis (≤ 10 cells/μL) was consistent with the distribution of optimal thresholds generated in 100 bootstrapped samples and fell within the IQR of these thresholds (ICU admission: median, 10 cells/μL [IQR, 10-23 cells/μL]; mechanical ventilation: Q1–median–Q3, median, 14, cells/μL [IQR, 10-42 cells/μL]). The mean area under the receiver operating characteristic curve across the 100 bootstrap datasets was 0.582 for ICU admission and 0.575 for mechanical ventilation, closely matching the areas under the receiver operating characteristic curve from the original data set (0.578 and 0.574, respectively).

### Comparison of Baseline Characteristics Between Eosinopenia and No Eosinopenia Groups

Among the 1,673 patients with available eosinophil counts, 549 patients (32.8%) had eosinopenia (≤ 10/μL) and 1,124 patients (67.2%) did not. Baseline demographic and clinical characteristics, including age, sex, BMI, laboratory values, and comorbidities, in the eosinopenia and no eosinopenia groups are provided in Table 3.

**Table 3:** Baseline Characteristics of the Eosinopenia vs No Eosinopenia Groups

Variable	Eosinopenia Group (≤ 10/μL; n = 549)	No Eosinopenia Group (> 10/μL; n = 1,124)	*P* Value
Age, y	66 (55-77)	67 (53-77)	.779^a^
Sample size	549 (100.0)	1,124 (100.0)	
Male sex	338 (61.6)	713 (63.4)	.484^b^
Sample size	549 (100.0)	1,124 (100.0)	
Weight, kg	75 (65.1-88.9)	77 (65.8-90)	.4458^a^
Sample size	521 (94.9)	1,097 (97.6)	
BMI, kg/m^2^	25.5 (23.27-29.15)	25.9 (22.6-29.4)	.481^a^
Sample size	516 (94.0)	1,094 (97.3)	
Neutrophil count, /nL	8.93 (5.33-15.14)	8.78 (6.13-12.04)	.843^a^
Sample size	161 (29.3)	178 (15.8)	
Creatinine, mg/dL	0.99 (0.81-1.20)	0.96 (0.77-1.27)	.394^a^
Sample size	547 (99.6)	1,111 (98.8)	
Hematocrit, %	38.2 (34.2-41.3)	37 (33.4-40.7)	.026^a^
Sample size	544 (99.1)	1,121 (99.7)	
CRP, mg/dL	12.57 (5.75-21.69)	10.27 (4.26-18.18)	< .001^a^
Sample size	546 (99.5)	1,109 (98.7)	
Asthma rate	40 (7.3)	100 (8.9)	.301^b^
Sample size	549 (100.0)	1,124 (100.0)	
COPD rate	95 (17.3)	237 (21.1)	.078^b^
Sample size	549 (100.0)	1,124 (100.0)	
Coronary heart disease rate	62 (11.3)	147 (13.1)	.345^b^
Sample size	549 (100.0)	1,124 (100.0)	
Cerebrovascular disease rate	306 (55.7)	673 (59.9)	.113^b^
Sample size	549 (100.0)	1,124 (100.0)	
Diabetes rate	98 (17.9)	231 (20.6)	.213^b^
Sample size	549 (100.0)	1,124 (100.0)	
Renal insufficiency rate	57 (10.4)	154 (13.7)	.060^b^
Sample size	549 (100.0)	1,124 (100.0)	
Liver cirrhosis rate	1 (0.2)	10 (0.9)	.115^b^
Sample size	549 (100.0)	1,124 (100.0)	
Dementia rate	6 (1.1)	13 (1.2)	1^b^
Sample size	549 (100.0)	1,124 (100.0)	
CURB-65 score	1 (0-2)	1 (0-2)	.127^a^
Sample size	434 (79.1)	878 (78.1)	
CURB score	1 (0-1)	0 (0-1)	.006^a^
Sample size	434 (79.1)	878 (78.1)	
CRB-65 score	1 (0-1)	1 (0-1)	.105^a^
Sample size	505 (92.0)	1,021 (90.8)	

Data are presented as No. (%) or median (interquartile range) unless otherwise indicated.

CRB-65 = confusion, respiratory rate, BP, and age ≥ 65 years; CRP = C-reactive protein; CURB = confusion, urea, respiratory rate, and BP; CURB-65 = confusion, urea, respiratory rate, BP, and age ≥ 65 years.

a Mann-Whitney *U* test.

b Fisher exact test.

### Comparison of Hospital Resource Use Between Eosinopenia and No Eosinopenia Groups

**Figure 2:**
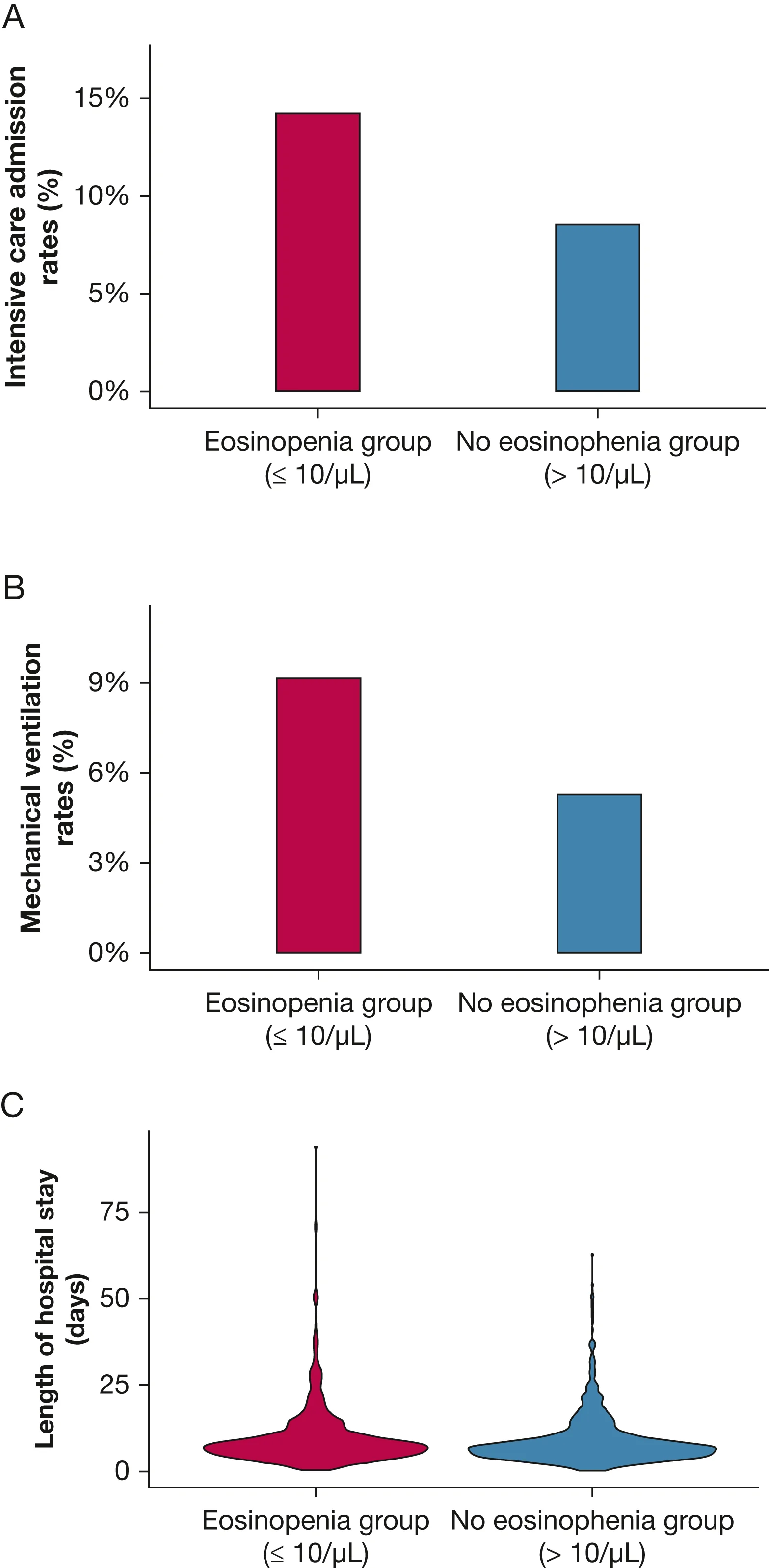
A, Bar graph showing ICU admission rates in patients with eosinopenia vs those without eosinopenia. Admission to the ICU occurred in 14.2% of patients with eosinopenia (n = 549) and 8.5% of those without eosinopenia (n = 1,124; *P* < .001; adjusted OR, 1.78; 95% CI, 1.22-2.69, multivariable regression analysis). B, Bar graph showing mechanical ventilation rates in patients with eosinopenia vs those without eosinopenia. Mechanical ventilation was required in 9.1% of patients with eosinopenia (n = 549) and in 5.2% of those without eosinopenia (n = 1,124; *P* = .003; adjusted OR, 1.82; 95% CI, 1.29-2.45, multivariable regression analysis). C, Graph showing length of hospital stays in patients with eosinopenia vs those without eosinopenia. Median length of stay was 10.2 days for patients with eosinopenia (n = 549) and 9.0 days for those without eosinopenia (n = 1,124; *P* = .013, multivariable regression analysis).

Eosinopenia (vs no eosinopenia) was associated with significantly higher intensive care admission rates (14.2% vs 8.5%, respectively; *P* < .001; adjusted OR, 1.78; 95% CI, 1.22-2.69) (Fig 2A) and mechanical ventilation rates (9.1% vs 5.2%; *P* = .003; adjusted OR, 1.82; 95% CI, 1.29-2.45) (Fig 2B). Furthermore, eosinopenia (vs no eosinopenia) was associated with longer hospital stays (10.2 days vs 9.0 days; *P* = .013) (Fig 2C). All comparisons of hospital resource use parameters between groups are presented in Table 4. No significant difference in in-hospital mortality was observed between the eosinopenia and no eosinopenia groups (*P* = .973).

**Table 4:** Hospital Resource Use in the Eosinopenia vs No Eosinopenia Groups

Variable	Eosinopenia Group (≤ 10/μL; n = 549)	No Eosinopenia Group (> 10/μL; n = 1,124)	*P* Value (MVA)	Adjusted OR (95% CI)	Adjusted Mean Difference (95% CI)
ICU admission rate	78 (14.2)	96 (8.5)	< .001	1.78 (1.22-2.69)	NA
Mechanical ventilation rate	50 (9.1)	59 (5.2)	.003	1.82 (1.29-2.45)	NA
Hospital stay, d	10.223 (5-11)	8.951 (5-10)	.013	NA	0.096 (0.02-0.17)
ICU stay, d	6.845 (2-9) (n = 77)	5.668 (1.75-6) (n = 96)	.232	NA	0.238 (–0.153 to 0.629)
Duration of mechanical ventilation, d	7.846 (2-10) (n = 49)	6.492 (1-5) (n = 59)	.170	NA	0.397 (–0.173 to 0.967)

Data are presented as No. (%) or median (interquartile range) unless otherwise indicated.

MVA = multivariable analysis considering eosinophil count as binary variable ≤ 10/μL vs > 10/μL, age, sex, creatinine, C-reactive protein, and hematocrit; NA = not applicable.

### Comparative Associations of Eosinopenia (≤ 10 cells/μL), Lymphopenia (< 0.724 cells/nL), and Dual Cytopenia With Hospital Resource Use

For ICU admission, all 3 binary markers were associated significantly with increased risk: eosinopenia (coefficient, 0.57; *P* < .001), lymphopenia (coefficient, 0.45; *P* = .004), and dual cytopenia (coefficient, 0.62; *P* = .001). For mechanical ventilation, significant associations were observed for eosinopenia (coefficient, 0.60; *P* = .003) and dual cytopenia (coefficient, 0.56; *P* = .018), whereas lymphopenia alone did not reach statistical significance (coefficient, 0.36; *P* = .066). For hospital length of stay, all 3 markers were associated significantly with longer duration: eosinopenia (coefficient, 0.10; *P* = .013), lymphopenia (coefficient, 0.13; *P* < .001), and dual cytopenia (coefficient, 0.19; *P* < .001). In terms of predictive performance, Youden’s index for ICU admission was 0.138 for eosinopenia, 0.124 for lymphopenia, and 0.113 for dual cytopenia; for mechanical ventilation, the corresponding values were 0.147, 0.109, and 0.091, respectively.

### Sensitivity Analysis Using IPW

The study included 1,260 patients with complete data for all propensity score covariates. After IPW adjustment, baseline characteristics were well balanced between the eosinopenia (≤ 10 cells/μL) and no eosinopenia groups, with all standardized mean differences of < 10%, indicating adequate covariate balance (e-Table 1). In the weighted regression models, lower eosinophil counts were associated significantly with increased rates of ICU admission (*P* < .001) and mechanical ventilation (*P* = .042).

### Sensitivity Analysis With Multiple Imputation

Using multiple imputation to account for missing data, lower eosinophil counts were associated significantly with increased risks of ICU admission (*P* = .004) and mechanical ventilation (*P* = .010).

## Discussion

In this prospective multicenter CAP cohort, lower eosinophil counts at hospital admission were associated with increased health care resource use, including higher ICU admission rates, greater mechanical ventilation needs, and prolonged length of stay. An eosinophil count threshold of ≤ 10 cells/μL was associated with a higher likelihood of requiring intensive and extended hospital care.

Previous studies have linked eosinopenia to worse outcomes in CAP, including increased mortality and sepsis risk.^10^^,^^11^ Our findings build on this evidence by showing that admission eosinophil counts are associated with health care use metrics. These results suggest that eosinophil counts not only may serve as a biomarker for clinical risk stratification, but also could help anticipate health care demands, thereby supporting more informed operational planning in acute care settings. Although eosinopenia was associated with higher ICU admission and mechanical ventilation rates, in-hospital mortality did not differ between groups. Eosinophil count seems to identify patients who are at higher risk of deterioration and require intensive care, rather than fully capturing determinants of death. Overall mortality was relatively low, limiting the power to detect mortality differences and possibly reflecting effective critical care in high-risk patients.

Acute infections and sepsis typically are marked by decreased eosinophil counts^15^ and neutrophilia. However, in this cohort, neutrophil counts were similar between the eosinopenia and no eosinopenia groups, suggesting that eosinopenia predicts hospital resource use independently of neutrophilia. Both eosinopenia^10^^,^^11^ and lymphopenia^14^ have been linked to higher rates of ICU admission and mortality in CAP. In this study, additional analyses showed that eosinopenia, lymphopenia, and dual cytopenia were related to greater ICU use and prolonged hospitalization; however, only eosinopenia (alone or combined with lymphopenia) consistently predicted the need for mechanical ventilation. These findings indicate that admission eosinophil count, particularly values of ≤ 10 cells/μL, provides clinically and operationally relevant information on hospital resource use and complement lymphocyte-based risk stratification. Although the association between eosinopenia and length of stay reached statistical significance, the absolute difference between groups was modest (approximately 1.2 days) and the continuous predictive power for length of stay was limited compared with ICU admission and mechanical ventilation.

In COVID-19 pneumonia, absolute eosinopenia has been associated with increased ventilatory support needs.^16^ Nonetheless, these findings may not be generalizable to CAP, which often is bacterial in origin.^17^ Standard diagnostic protocols typically do not allow for rapid identification of the causative pathogen at the time of hospital admission, creating a need for a readily available biomarker to predict health care resource requirements early in the disease course, before microbiological results become available. Our findings indicate that admission eosinophil counts—a standard component of routine blood tests—can help to identify patients with CAP at higher risk of intensive hospital resource use independent of microbial cause and with less complexity than composite severity scores requiring multiple clinical and laboratory parameters.

Known predictors of ICU admission and prolonged hospitalization in CAP include vital signs, radiographic findings, and clinical scoring systems such as the Pneumonia Severity Index or quick Sequential (Sepsis-Related) Organ Failure Assessment score.^18^, ^19^, ^20^, ^21^, ^22^, ^23^ Glucocorticoid therapy is an important factor that suppresses eosinophil counts.^24^ However, its role in CAP management remains inconclusive,^25^^,^^26^ with trials reporting mixed effects on mortality.^27^, ^28^, ^29^ Glucocorticoid exposure was handled through stratified analyses, rather than incorporated as a covariate, because it functions both as a confounder and potential mediator of eosinopenia.

Importantly, our findings indicate that eosinophil counts of ≤ 10 cells/μL offer prognostic value independent of glucocorticoid use, because the association between eosinophil counts and ICU admission persisted both in patients who received systemic corticosteroids and in those who did not. This finding suggests that eosinopenia was associated with ICU admission regardless of whether the eosinopenia resulted from glucocorticoid-induced suppression or developed through other pathogenic mechanisms, suggesting that eosinophil count provides prognostic information independent of glucocorticoid exposure.

An eosinophil count threshold of ≤ 10 cells/μL best discriminated between higher and lower risk of ICU admission and mechanical ventilation, and high negative predictive values indicate that patients with eosinophil counts of > 10/μL are at comparatively lower risk. However, the modest sensitivity and residual risk in patients with eosinophil counts higher than this threshold underscore that eosinophil counts alone are insufficient to rule in or rule out definitively the need for ICU-level care and should be integrated into broader risk stratification frameworks to support hospital resource planning.

Eosinophil count is an inexpensive, readily available, and routinely measured laboratory parameter. Incorporating eosinophil counts into hospital resource use models could support timely decisions on patient triage, ICU allocation, and discharge suitability. This, in turn, may support more efficient allocation of hospital resources in response to substantial pressure on health care systems worldwide.^12^

Future studies should evaluate whether incorporating eosinophil counts into existing prognostic models improves the prediction of hospital resource needs in CAP, should validate our findings in independent patient cohorts, and should elucidate mechanisms underlying eosinopenia in CAP, including potential variation by microbial cause. Interventional studies could assess whether eosinophil-guided triage strategies lead to improved clinical or operational outcomes.

### Strengths and Limitations

Strengths of this study include its prospective multicenter design, large sample size, and standardized data collection, which enhance the generalizability and robustness of the findings. Adjustment for key confounders further strengthens the validity of the results. Multiple imputation and IPW were conducted for ICU admission and mechanical ventilation, and these sensitivity analyses confirmed that the observed associations between lower eosinophil counts and these outcomes remained statistically significant after accounting for missing data and potential confounding. Limitations include missing data on adjustment covariates, specifically, eosinophil count, creatinine, C-reactive protein, and hematocrit and pneumonia severity scores like pneumonia severity index; systolic BP, multilobar infiltrates, albumin, respiratory rate, tachycardia, confusion, oxygen, and pH; Infectious Diseases Society of America and American Thoracic Society minor criteria; time from symptom onset to hospital admission; and vaccination status, which may affect outcomes. The lack of complete severity scores limited adjustment for all potential confounders, which may introduce residual confounding despite multivariable control for key laboratory and demographic variables. Moreover, information on mortality beyond hospital discharge (eg, 30-day mortality) was not captured on a regular basis, so analyses were limited to in-hospital mortality. Finally, the limited availability of microbiologically confirmed cases restricted pathogen-specific analyses.

## Interpretation

In this prospective multicenter cohort study, lower eosinophil counts at hospital admission were associated with increased hospital resource use in patients with CAP. As an accessible and early biomarker, eosinophil count may aid timely clinical decision-making for triage, ICU admission, and discharge planning, thereby supporting more efficient hospital resource allocation.

## Funding/Support

This work was supported by the 10.13039/501100002347German Federal Ministry of Education and Research [Grant 01KI07145], the 10.13039/501100010564Deutsches Zentrum für Lungenforschung (DZL; German Center for Lung Research), and the LOEWE research cluster “Health Affected by Climate Change and Air Pollution—Pathophysiology and Regional Management (HABITAT).” The investigators of this scientific work acknowledge the CAPNETZ STIFTUNG and the CAPNETZ Study group for project support regarding the use of biomaterials and clinical data (project reference numbers: 2018-06-11-F5JI and 2024-07-11-CHV6). CAPNETZ is a multidisciplinary approach to improved understanding and treatment of patients with CAP (https://capnetz.de/en/home/).

## Financial/Nonfinancial Disclosures

The authors have reported to *CHEST* the following: B. C. W. reports leadership as chair of the Scientific Advisory Board, German Lung Foundation; participation in an advisory board with AstraZeneca; financial support for attending meetings, travel, or both from Universität Marburg/UKGM; and participation in an expert meeting organized by INSMED, for which no financial support or reimbursement was accepted. P. Ahnert reports support from the German Federal Ministry for Research, Technology and Space (BMFTR, formerly BMBF; Grants PROGRESS: 01KI07113 and 01KI1010I; CAPSyS: 01ZX1304A; SYMPATH-1 and -2: 01ZX1906B and 01ZX2206B; ScaDS.AI: 01IS18026B; German Center for Lung Research [DZL]: 82DZLJ19A2, 82DZLJ19B2, and 82DZLJ19C2; payments were made to the institution that paid part of the salary for P. A. from the various grants and otherwise made the project possible); an international patent application (PCT/EP2024/080280: “Novel biomarkers for prognosis of community acquired pneumonia (CAP)”); and leadership as speaker of the NAKO Expert Group on infectious diseases and the working group genetic epidemiology of the German Society for Epidemiology. M. W. reports grants from the Bundesministerium für Bildung und Forschung (BMBF; Federal Ministry of Education and Research); Deutsche Forschungsgemeinschaft (DFG; German Research Foundation); Gemeinsamer Bundesausschuss (G-BA; The Federal Joint Committee); Bund; Bundesministerium für Gesundheit (BMG; Federal Ministry of Health); Biotest, Pantherna, Aptarion. M. W. P. reports a grant from the German Federal Ministry for Education and Research for the present manuscript; grants or contracts from Pfizer; consulting fees to the intuition from Pfizer, MSA, Sanofi, Janssen, GSK, AstraZeneca, Shionogi, and Infectopharm; payment or honoraria for lectures, presentations, speakers bureaus, manuscript writing, or educational events from Pfizer, MSA, Sanofi, Janssen, GSK, AstraZeneca, Shionogi, Infectopharm, BioMérieux, and Sanofi; support for attending meetings, travel, or both from Pfizer and MSD; patents planned, issued, or pending with bioactive glass element; participation on a data safety monitoring board or advisory board with BioMérieux and Sanofi; and leadership as president Paul Ehrlich Society for Antiinfective Chemotherapy and on the board of directors of CAPNETZ and the German Sepsis Society. U. M. Z. reports grants or contracts from Bavarian State Office for Health and Food Safety [Grant K1-2497-GLB-20-V4], Bavarian States Ministry of Science and Art (grants and personal fees), and Max Aicher foundation (grants and personal fees), and leadership of Cfi-aktiv Mukoviszidose Selbsthilfe Südbayern e.V. (unpaid). T. G. reports grants or contracts from Grifols to institution for AATD laboratory; consulting fees from AstraZeneca, Berlin-Chemie, Boehringer-Ingelheim, Chiesi, CSL-Behring, Grifols, GSK, Mundipharma, Novartis, and Takeda; payment or honoraria for lectures, presentations, speakers bureaus, manuscript writing, or educational events from AstraZeneca, Berlin-Chemie, Boehringer-Ingelheim, Chiesi, CSL-Behring, Grifols, GSK, Mundipharma, Sanofi, and Takeda; support for attending meetings or travel from AstraZeneca, Berlin-Chemie, Chiesi, CSL-Behring, Grifols, GSK, Novartis, and Sanofi; and participation on a data safety monitoring board or advisory board for AstraZeneca, Berlin-Chemie, Boehringer-Ingelheim, Chiesi, CSL-Behring, Grifols, GSK, Mundipharma, Novartis, Sanofi, and Takeda. T. B. reports funding via the LOEWE program from the State Hessia in Germany. P. Alter reports support of COSYCONET (unrestricted grants) from GlaxoSmithKline GmbH & Co. KG, Chiesi GmbH, AstraZeneca GmbH, and Sanofi Aventis GmbH; consulting fees for advisory board participation from Sanofi-Aventis Deutschland GmbH; payments or honoraria for a presentation on hypertension and heart failure from StreamedUp GmbH; and support for attending meetings, travel, or both from Sanofi-Aventis Deutschland GmbH and Boehringer Ingelheim GmbH. G. R. reports consulting (personal) fees from Astra Zeneca, Atriva, Boehringer Ingelheim, GSK, Insmed, MSD, Sanofi, Novartis, and Pfizer for consultancy during advisory board meetings; payment or honoraria (personal fees) for lectures from Astra Zeneca, Berlin Chemie, BMS, Boehringer Ingelheim, Chiesi, Essex Pharma, Grifols, GSK, Insmed, MSD, Roche, Sanofi, Solvay, Takeda, Novartis, Pfizer, and Vertex; and leadership as chairman of the CAPNETZ executive board (unpaid). C. F. V. reports grants or contracts to the institution from German Ministry of Education and Science (BMBF), AstraZeneca, Boehringer Ingelheim, Chiesi, CSL Behring, GlaxoSmithKline, Grifols, and Novartis; personal consulting fees from Aerogen, AstraZeneca, Boehringer Ingelheim, CSL Behring, Chiesi, GlaxoSmithKline, Insmed, Menarini, Novartis, Nuvaira, Roche, and Sanofi; and personal payments or honoraria for lectures, presentations, speakers bureaus, manuscript writing, or educational events from Aerogen, AstraZeneca, Boehringer Ingelheim, CSL Behring, Chiesi, GlaxoSmithKline, Insmed, Menarini, Novartis, Roche, and Sanofi. B. S. reports public research funding (unlimited) to Marburg University without influence on the study from the German Federal Ministry for Education and Research (BMBF): medical informatics initiatives MIRACUM and CALM-QE as well as German Center for Lung Research (DZL), PermedCOPD, and German Federal Ministry for Health: PROGRESS PostCOVID cohort; and grants or contracts from CSL Behring (research collaboration with Marburg University; no relation to this study). None declared (Q. W., W. B., M. K., R. R., S. A. K., N. S., C. W., J. R., A. M.).

## Supplementary Data

e-Online Data

Audio
